# β-Cyclodextrin-based nanoassemblies for the treatment of atherosclerosis

**DOI:** 10.1093/rb/rbae071

**Published:** 2024-06-17

**Authors:** Weihong Ji, Yuanxing Zhang, Weichen Shao, Ranjith Kumar Kankala, Aizheng Chen

**Affiliations:** Institute of Biomaterials and Tissue Engineering, Huaqiao University, Xiamen, Fujian 361021, PR China; Fujian Provincial Key Laboratory of Biochemical Technology, Huaqiao University, Xiamen, Fujian 361021, PR China; The Institute of Forensic Science, Xiamen Public Security Bureau, Xiamen, Fujian 361104, PR China; Institute of Biomaterials and Tissue Engineering, Huaqiao University, Xiamen, Fujian 361021, PR China; Fujian Provincial Key Laboratory of Biochemical Technology, Huaqiao University, Xiamen, Fujian 361021, PR China; Institute of Biomaterials and Tissue Engineering, Huaqiao University, Xiamen, Fujian 361021, PR China; Fujian Provincial Key Laboratory of Biochemical Technology, Huaqiao University, Xiamen, Fujian 361021, PR China; Institute of Biomaterials and Tissue Engineering, Huaqiao University, Xiamen, Fujian 361021, PR China; Fujian Provincial Key Laboratory of Biochemical Technology, Huaqiao University, Xiamen, Fujian 361021, PR China

**Keywords:** β-cyclodextrin, nanoassemblies, drug delivery, inflammation, atherosclerosis

## Abstract

Atherosclerosis, a chronic and progressive condition characterized by the accumulation of inflammatory cells and lipids within artery walls, remains a leading cause of cardiovascular diseases globally. Despite considerable advancements in drug therapeutic strategies aimed at managing atherosclerosis, more effective treatment options for atherosclerosis are still warranted. In this pursuit, the emergence of β-cyclodextrin (β-CD) as a promising therapeutic agent offers a novel therapeutic approach to drug delivery targeting atherosclerosis. The hydrophobic cavity of β-CD facilitates its role as a carrier, enabling the encapsulation and delivery of various therapeutic compounds to affected sites within the vasculature. Notably, β-CD-based nanoassemblies possess the ability to reduce cholesterol levels, mitigate inflammation, solubilize hydrophobic drugs and deliver drugs to affected tissues, making these nanocomponents promising candidates for atherosclerosis management. This review focuses on three major classes of β-CD-based nanoassemblies, including β-CD derivatives-based, β-CD/polymer conjugates-based and polymer β-CD-based nanoassemblies, highlighting a variety of formulations and assembly methods to improve drug delivery and therapeutic efficacy. These β-CD-based nanoassemblies exhibit a variety of therapeutic mechanisms for atherosclerosis and offer systematic strategies for overcoming barriers to drug delivery. Finally, we discuss the present obstacles and potential opportunities in the development and application of β-CD-based nanoassemblies as novel therapeutics for managing atherosclerosis and addressing cardiovascular diseases.

## Graphical Abstract

**Figure:**
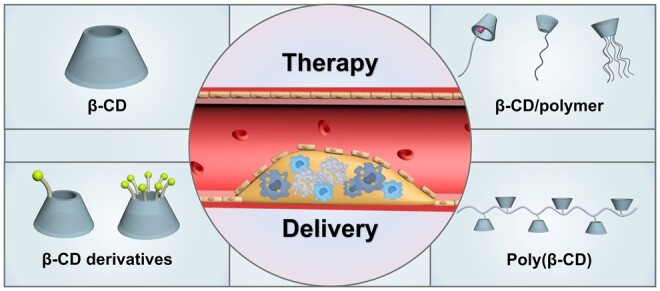

## Introduction

Cardiovascular and cerebrovascular diseases (CVDs) have become the leading cause of death worldwide [1–3]. In particular, atherosclerosis has got widespread attention because it has greatly increased the incidence of myocardial infarction, cerebral stroke and other serious CVDs [4–6]. Due to dietary modifications and global population aging, atherosclerosis has become a common health problem. It is the result of the interaction of multiple genetic and environmental risk factors. Hyperlipidemia caused by high blood lipid or cholesterol is the biggest risk factor for atherosclerosis [7]. The onset and development of atherosclerosis is mostly dependent on the buildup of lipoproteins, particularly low-density lipoprotein-cholesterol (LDL-C) in the arterial wall [8]. The LDL-C penetrates damaged endothelial cells into the intima and is phagocytosed by macrophages to form foam cells, participating in the progression of inflammation and atherosclerotic plaque. Lipid deposition and the appearance of cholesterol crystals (CCs) in atherosclerotic lesions have been identified as one of the predominant causes of atherosclerotic plaque inflammation [9, 10]. Meanwhile, inflammation can accelerate vascular hyperplasia, leading to further plaque development and forming a vicious circle [11–13]. Therefore, inflammation is also a risk factor that cannot be ignored for the development of atherosclerosis [14, 15].

**Figure 1.**
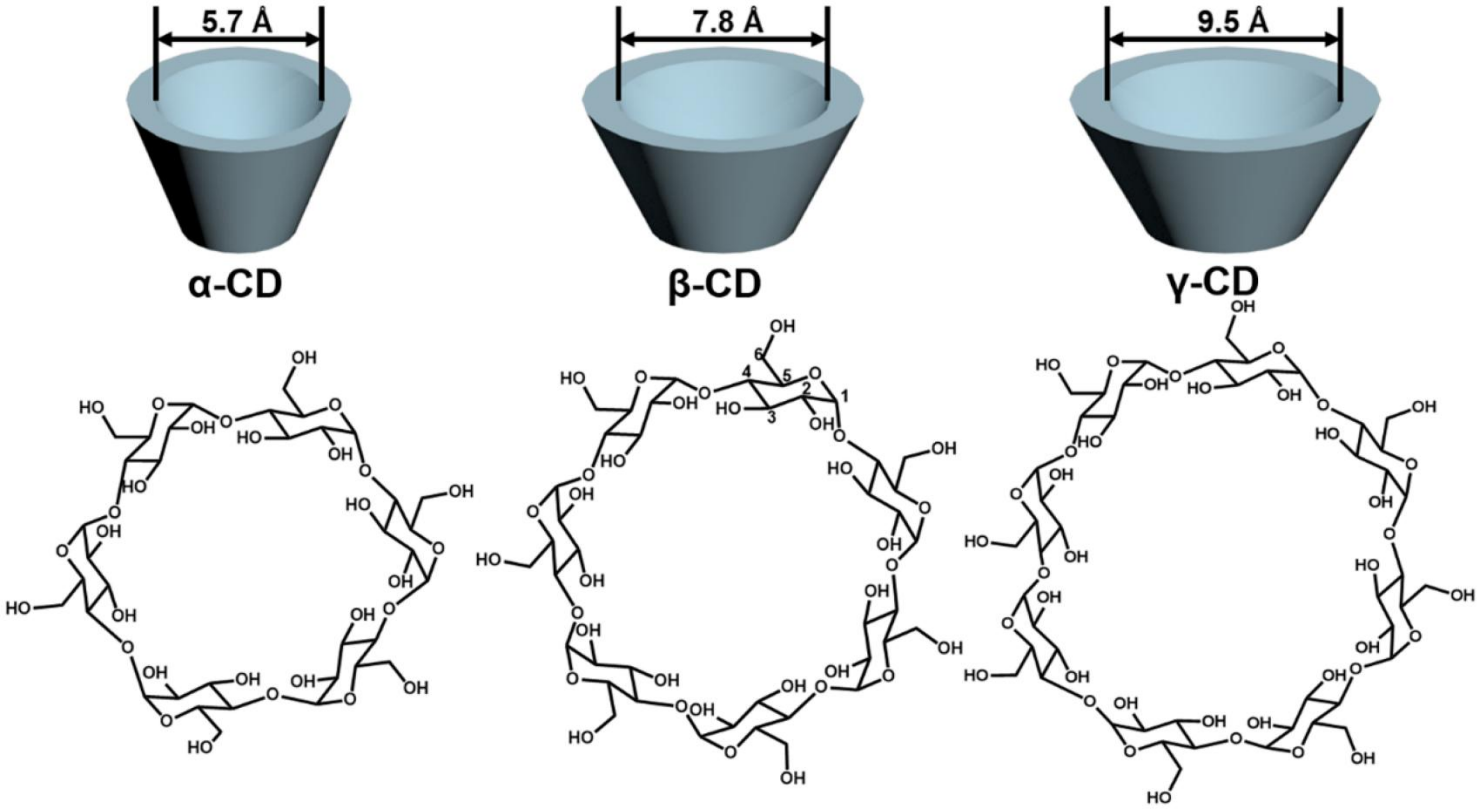
The structure and conformation of α-, β- and γ-CD.

Cyclodextrins (CDs) are a group of cyclic oligosaccharides that are made up of large rings of glucose subunits connected by α-1,4-glucoside bonds [16]. The most typical CDs include α-, β- and γ-CD, which contain many glucose monomers with six, seven, and eight units in the ring to form a truncated cone shape (Figure 1) [17]. The diameters of their internal cavity are 5.7, 7.8 and 9.5 Å, respectively [18]. CDs owe their water solubility to their numerous hydroxyl groups. Among α-, β- and γ-CD, their water solubility percentages are 14%, 2% and 23% w/w, respectively [18]. β-Cyclodextrin (β-CD) exhibits lower solubility compared to α- and γ-CD due to internal hydrogen bonding involving secondary hydroxyl groups, which rigidifies its shape. Despite being less soluble, β-CD possesses a cavity size and shape suitable for forming complexes with many aromatic ring drugs [19, 20]. In comparison to β-CD, α-CD can usually only bind a single organic hydrocarbon chain due to its smaller cavity. γ-CD with a bigger cavity can bind larger guest molecules, but the collapsing characteristics of the internal cavity and the higher production cost limit its application [21, 22]. Overall, β-CD stands out as the most commonly utilized CD because of its moderate cavity and low production cost. In particular, β-CD with a special structure can bind cholesterol and CCs, offering advantages in the treatment of atherosclerosis.

## Structure and properties of β-CD

β-CD consists of seven glucose units, each with two secondary hydroxyl groups at positions C2 and C3 and a primary hydroxyl group at position C6 (Figure 1) [16]. The two secondary hydroxyl groups at positions C2 and C3 are located at the broad rim, and the primary hydroxyl group at position C6 is located at the narrow rim. Because of its truncated conical structure, which consists of an inner hydrophobic surface and an outer hydrophilic surface, β-CD has good binding characteristics for hydrophobic drug molecules or amphipathic molecules with a hydrophobic portion, such as cholesterol [23–25]. Additionally, the inner diameter of the β-CD cavity is optimal for binding cholesterol and CCs. Therefore, β-CD has been widely used for lowering cholesterol levels and removing insoluble CCs [26, 27]. However, the interactions between C2/C3 secondary hydroxyl groups of adjacent glucose units limit the water solubility of β-CD to a considerable extent. The formation and accumulation of CD-cholesterol or CD-CCs complexes may lead to further decline in solubility and even precipitate in aqueous systems, which may be a significant factor in causing acute renal toxicity by intravenous administration [28, 29]. Consequently, β-CD may also easily and efficiently extract plasma membrane-bound cholesterol, which can greatly induce hemolysis and increase ototoxicity [30, 31]. Therefore, β-CD has been chemically modified to improve solubility and biocompatibility for clinical applications.

To further meet drug delivery needs, β-CD derivatives have been developed and applied. All chemical modifications occur on the hydroxyl groups of β-CD [32, 33]. Experiments have shown that the hydroxyl group at position C6 exhibits good reactivity due to its small steric hindrance and nucleophilic property [21]. The hydroxyl group located at position C2 is the most acidic. It can form reactive oxyanions, whereas the hydroxyl group at position C3 is the most inaccessible and the least reactive due to its active steric hindrance [34]. Highly reactive reagents can react with all hydroxyl groups indiscriminately, while less reactive reagents can react preferentially with the C6 hydroxyl group. Various modified-β-CDs, such as sulfobutylether-β-CD (SBE-β-CD), hydroxypropyl-β-CD (HP-β-CD) and methyl-β-CD (M-β-CD), have been widely used in numerous applications [35, 36]. Although the cost of derivatives has increased to a certain extent, the water solubility of β-CD derivatives has been greatly improved, which has greatly expanded the scope of application of β-CD. Notably, SBE-β-CD, a polyanionic β-CD derivative that has undergone thorough safety evaluations, has been utilized in a variety of Food and Drug Administration (FDA) approved drugs as well as in preclinical and clinical studies [37]. Captisol^®^ (SBE-β-CD) has been manufactured in pharmaceutical-related products for oral, ophthalmic, intravenous and subcutaneous administration, which can significantly improve the solubility, stability, and bioavailability of insoluble drugs [38]. Another example is HP-β-CD, which is also an FDA-approved substance to dissolve and load numerous hydrophobic drugs for medicinal applications in humans [39]. Sporanox^®^, an HP-β-CD-itraconazole injection, has significantly increased the solubility of itraconazole from 1 ng/ml to 10 mg/ml [40]. Therefore, β-CD derivatives hold great potential in the treatment of atherosclerosis.

**Figure 2.**
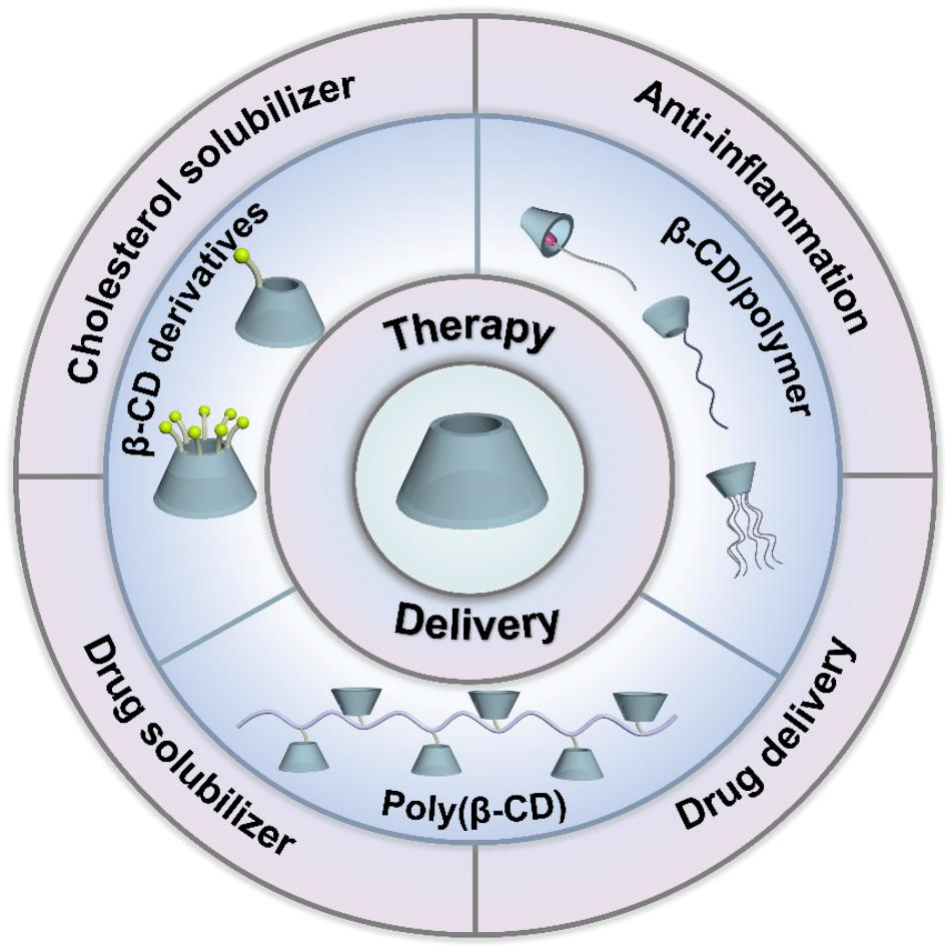
A schematic overview of β-CD-based nanoassemblies for the treatment of atherosclerosis.

In recent years, the development of nanomedicines has emerged as a prominent area in medication innovation [41]. Due to its good biosafety and unique structure, β-CD has the potential to significantly enhance nanoparticles formulation performance [42–44]. β-CD-based nanoassemblies have been used for delivering anticancer drugs, antifungal agents and anti-inflammatory drugs, which can increase the solubility and specificity of drugs, and improve pharmacokinetics and bioavailability in drug delivery [18, 45]. Moreover, β-CD and its derivatives exhibit the ability to interact with certain toxins and cholesterol, indicating potential applications in the management of detoxification and hypercholesterolemia [46, 47]. And it has been proved that β-CD and its derivatives can exert anti-atherogenic effects. These effects include increasing cholesterol and CCs solubility, promoting the removal of cholesterol and CCs from foam cells, reducing inflammation in blood vessels, reducing plaque size and promoting plaque regression [48–50]. Given the promising potential of β-CD in the treatment and drug delivery for atherosclerosis, this review will focus on β-CD-based nanoassemblies for anti-atherosclerosis. Therefore, not only the therapeutic effect of CD is involved, but also the use of β-CD-based nanoassemblies for drug delivery is emphasized because of the size and shape of the β-CD cavity, which can form complexes with many aromatic ring drugs for drug delivery [18, 51]. Specifically, β-CD derivatives-based, β-CD/polymer-based, and polymer β-CD (poly(β-CD))-based nanoassemblies will be reviewed in sequence (Figure 2). The challenges and future development of β-CD-based nanoassemblies for anti-atherosclerosis are presented finally.

## Methodology

A search for relevant literature was conducted in the PubMed and Web of Science databases. The search was not restricted by date and included the following terms: ‘β-cyclodextrin’ AND ‘atherosclerosis’ AND ‘supramolecular nanoassembly’ OR ‘drug delivery’ OR ‘therapy’ OR ‘cholesterol’ OR ‘inflammation’. The search was limited to English language publications.

### β-CD-based nanoassemblies for anti-atherosclerosis

Table 1 lists three major classes of β-CD-based nanoassemblies, including those based on small molecule β-CD derivatives, β-CD/polymer conjugates and poly(β-CD). Owing to the corresponding β-CD components, the nanoassemblies for drug delivery can be stably constructed for the treatment of atherosclerosis.

**Table 1.** Summary of β-CD-based nanoassemblies for drug delivery aimed at treating atherosclerosis

Class	Nanoassemblies	Components	Drug	Effects	References
Small molecule β-CD derivatives-based	RAP/Ac-bCD180	Acetalated β-CD	Rapamycin	Improved drug solubility; sustaining drug release	[52]
	Ac-bCD and Ox-bCD	Acetalated β-CD and ROS-sensitive β-CD	Rapamycin	Improved drug solubility; released drug in pH and ROS-responsive manner	[53]
	LCD	CDI-activated β-CD	Luminol	Excellent safety profile; effective anti-inflammatory	[54]
	CSNP	M-β-CD	Simvastatin	Released statins and depleted cholesterol in the plaque micro-environment	[55]
β-CD/polymer-based	AAM	Acetalated-CD, DSPE-PEG/DSPE-PEG-cRGDfK, PEI	Anti-miR33	Targeted delivery; pH-responsive drug release	[56]
	TPCP	Tempol and phenylboronic acid pinacol ester functionalized-β-CD (TPCD), DSPE-PEG	TPCD	Targeted therapy; scavenged broad-spectrum ROS	[57]
	TPCDP@PMM	Two-photon fluorophore modified-CD, PMEMA-PMPC	Prednisolone	Improved drug solubility; controlled drug release; theranostics	[6]
	PLCDP@PMH	LXR-L-linked β-CD, MMP-9 responsive peptide, PMEMA, oxHA	Prednisolone and photoacoustic imaging agent	Improved drug solubility; controlled drug release; targeted theranostics	[58]
	MM@MTX NPs	MPEG-β-CD, macrophage membranes	Methotrexate	Improved drug solubility; extended blood retention time; targeted delivery	[59]
Poly(β-CD)-based	Cy7-CDP	β-CD polymer	β-CD	Extended blood retention time; improved plaque accumulation; decreased ototoxicity; removed lipids	[60]
	pCD/pBM-SNA	Poly (β-CD), poly-benzimidazole, dextran sulfate	M-β-CD	Targeted plaque delivery; pH-sensitive switch; removed lysosomal CCs	[61]

### β-CD derivatives-based nanoassemblies

The chemical modification of β-CD with different functional groups can change their characteristics, such as their capacity to bond with cholesterol. Research has shown that comparing the interactions between β-CD derivatives and cholesterol is crucial to choose the most suitable derivative for effectively removing cholesterol. The HP-β-CD/cholesterol and SBE-β-CD/cholesterol complexes exhibited moderate intermolecular hydrogen bonds, but the M-β-CD/cholesterol complex displayed weak intermolecular hydrogen bonds [62]. The fact that the complexation energy of HP-β-CD/cholesterol complex was lower than that of the three complexes consisting of β-CD, M-β-CD and SBE-β-CD with cholesterol, respectively, offered more proof that the hydroxypropyl substituent of HP-β-CD was the most successful in boosting the complexation of cholesterol with the derivative HP-β-CD [62]. The following factors can also affect the binding of β-CD derivatives to cholesterol, such as the hydrophilicity and electability of functional groups, and the degree and position of substitution on the CD molecule.

In theory, each β-CD molecule has seven glucose residues and can form a series of homologs with an average degree of substitution ranging from 1 to 21. In practice, during the synthesis process, each substitution form does not occur with equal probability. Different catalysts can cause substitution to occur specifically at the 2-, 3- or 6-hydroxyl (-OH) positions. Due to factors such as steric hindrance, the probability of functional groups forming at the same position on different glucose rings of β-CD is higher when the positions are more dispersed, and it also depends on the specific experimental conditions during synthesis [21]. Therefore, SBE-β-CD, HP-β-CD and other CD derivatives with a certain average degree of substitution are actually a mixture of different substitution positions.

A variety of hydrophobic drug molecules with an aromatic ring can be encapsulated into the β-CD cavity, such as statins, steroids, flavonoids and phenolic compounds [63]. However, the solubility of β-CD in water is constrained because of the interaction of internal hydrogen bonds, which restricts its use in parenteral products. The β-CD derivatives such as M-β-CD, HP-β-CD and SBE-β-CD have been used and developed into corresponding marketed formulations to overcome this problem [18, 64]. Recently, β-CD derivatives have been incorporated into a range of nano-sized formulations, which have the characteristics of excellent drug loading capacity, low toxicity and long blood circulation time, and show great potential in terms of atherosclerosis treatment [65–67]. Dou and colleagues [52] designed an acetalated β-CD (Ac-β-CD) material-based nanocarrier for delivering the anti-atherosclerotic drug rapamycin sustainably. Compared to natural β-CD, Ac-β-CD has improved solubility in water and common solvents like dichloromethane, ethanol or acetone. By utilizing a standard emulsion technique, Ac-β-CD could be processed into nanoparticles loaded with hydrophobic drugs, such as rapamycin. Ac-β-CD with well-defined pH-sensitive degradability could achieve sustained release of rapamycin and undergo degradation into water-soluble and non-acidic parent compounds. Both *in vitro* and *in vivo* studies have confirmed that rapamycin/Ac-β-CD nanoparticles have good biocompatibility and therapeutic advantages against atherosclerosis. Moreover, Dou *et al.* also synthesized a reactive oxygen species (ROS)-sensitive β-CD material (Ox-β-CD) by chemical modification of β-CD with 1,1′-carbonyldiimidazole (CDI)-activated phenylboronic acid pinacol ester, which was formed into a ROS-responsive nanoparticle loaded with rapamycin. After intraperitoneal delivery in ApoE^−/−^ mice, the β-CD derivative-based NP significantly enhanced the stability of atherosclerotic plaques [53].

**Figure 3.**
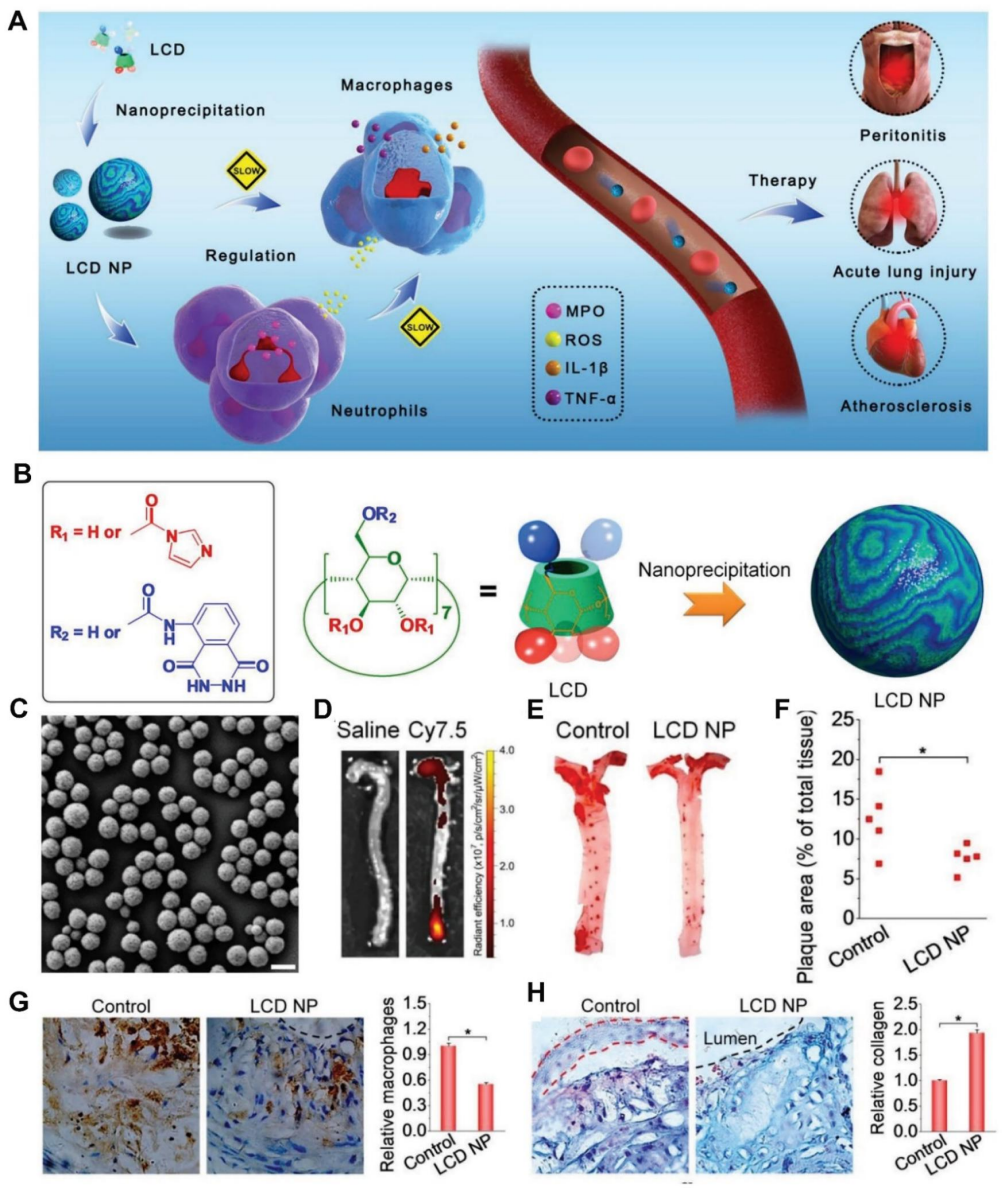
Engineered LCD NP using β-CD against inflammatory diseases. (**A**) Schematic illustration of a nanotherapy for inflammatory diseases. (**B**) Schematic illustration of the LCD NP preparation process. (**C**) SEM images of LCD NP. Scale bar: 200 nm. (**D**) Images of LCD NP labeled with Cy7.5 in the aorta *ex vivo*. (**E**) Representative images of ORO-stained aortas and (**F**) the quantification of the plaque area. (**G**) Representative images and quantification of the macrophage of aortic root sections. (**H**) Representative images and quantification of the collagen level of aortic roots. Adapted with permission from Ref [54].

Inflammation is a common culprit of various chronic and acute inflammatory diseases, such as metabolic diseases, infectious diseases, neurodegenerative diseases and cardiovascular diseases [68–72]. Guo *et al.* [54] engineered an anti-inflammatory nanoparticle using β-CD derivatives for inflammatory diseases, including atherosclerosis, acute lung injury and peritonitis (Figure 3A). Specifically, β-CD was first activated *via* CDI. Then, a β-CD conjugated with luminol (LCD) material was prepared by a nucleophilic reaction between CDI-activated β-CD and luminol, and LCD nanoparticle (LCD NP) was finally constructed by a nanoprecipitation approach (Figure 3B). The practical, low-cost and scalable LCD NP with nano-size showed good dispersion in deionized water (Figure 3C). The LCP NP was intraperitoneally or intravenously administered in atherosclerosis, acute lung injury and peritonitis mice models, and exhibited anti-inflammatory efficacies. As depicted in Figure 3D, significant focal fluorescent signals of Cy7.5-labeled LCD NP appeared in the aortic arch of ApoE^−/−^ mice, suggesting that the NP could accumulate within atherosclerotic plaques. The LCD NP significantly reduced plaque areas of the aorta in ApoE^−/−^ mice (Figure 3E and F). Literature has shown that increased plaque vulnerability is accompanied by an increase in macrophages and a decrease in collagen [73–76]. Immunohistochemistry revealed that LCD NP significantly reduced the macrophage count and increased the collagen content surrounding plaques (Figure 3G and H). LCD NP could effectively enhance the stability of atheromatous lesions and act as an efficient anti-inflammatory nanotherapy.

In comparison to natural β-CD, the highly water-soluble β-CD derivatives like M-β-CD can further enhance the aqueous solubility of hydrophobic molecules such as cholesterol and hydrophobic drugs [77–79]. Therefore, β-CD derivatives have often been used for drug delivery and controlled release, as well as for simultaneous lipid removal in the cholesterol-rich plaque. Statins, such as simvastatin, are commonly utilized as cholesterol-lowering medications to prevent and combat atherosclerosis and other hyperlipidemia diseases [80, 81]. Kim *et al.* [55] constructed an inclusion complex of M-β-CD and simvastatin (CD-ST) capable of driving the exchange of cargo within the cavity through affinity. The CD-ST complex as a core was coated with a lipid layer to prepare cargo-switching nanoparticles (CSNP). In ApoE^−/−^ mice, intravenously injected CSNP could successfully target the plaque *via* passive targeting, which could enable preferential accumulation of CD and simvastatin in plaque and avoid severe hearing loss. Subsequently, CSNP could release simvastatin and scavenge cholesterol in the cholesterol-rich plaque environment, which was attributed to the stronger affinity of CD with cholesterol than simvastatin. As a result, CSNP composed of M-β-CD and simvastatin demonstrated superior anti-atherogenic efficacy compared to conventional liposomal drug delivery systems. In summary, β-CD derivatives inherit the unique structure and properties of β-CD, enabling effective integration with hydrophobic structures for drug delivery and relief of pathology. Moreover, structural reconstruction can significantly improve the solubility of β-CD, thereby enhancing efficacy and reducing side effects.

### β-CD/polymer-based nanoassemblies

To enhance drug delivery efficiency, polymers are frequently introduced to form nanoparticles together with β-CD or β-CD derivatives [51, 82, 83]. The addition of polymers can increase the following functions, including expanding the range of cargoes loaded, such as gene drugs and imaging agents, improving plaque-targeting accumulation, achieving responsive degradation and controlled release and integrating treatment with diagnosis [84–86]. The interactions between CDs and polymers primarily involve non-covalent and covalent interactions. Hence, β-CD/polymer-based nanoassemblies are discussed further.

### β-CD/polymer noncovalent conjugates-based nanoassemblies

Nucleic acid drugs, such as antisense oligonucleotide (ASO), small interfering RNA (siRNA), microRNA (miRNA) and messenger RNA (mRNA), have undergone rapid development in treating various diseases, including antiviral, anti-tumor, cardiac and metabolic diseases, liver diseases and many rare diseases [87, 88]. In 2020, the first PCSK9-targeted siRNA drug (Inclisiran) was approved by the FDA for the treatment of hypercholesterolemia, which has also demonstrated a significant reduction in LDL cholesterol levels in atherosclerosis patients [89]. The specialized GaINAc delivery system is used for Inclisiran to achieve targeted delivery to the liver. Limited by the immaturity of delivery technology, the current siRNA targets are still mainly limited to liver tissues, whether based on liposome or GaINAc delivery system [90]. For atherosclerosis, the formation and development of plaque are important events in disease progression, so targeting plaque delivery can improve drug therapeutic effectiveness. Li *et al.* [56] developed an ASO against miRNA-33 (anti-miR33) nanotherapy (AAM NP) to improve cholesterol efflux and modulate macrophage polarization for the treatment of atherosclerosis. In order to improve load capacity and transfection efficiency, the cationic material polyethyleneimine (PEI) was introduced to load anti-miR33 by electrostatic adsorption. Besides, poly(ethylene glycol) (PEG) chains were additionally adorned with a peptide ligand cRGDfK to achieve a targeted delivery. The pH-responsive and biodegradable material acetalated-CD was prepared and used to construct the core of NPs. The acetalated-CD could be selectively hydrolyzed under the mildly acidic condition of endosomes/lysosomes, which could trigger anti-miR33 escape and release anti-miR33 in the cytoplasm. AMM NP successfully achieved enrichment in plaques and target cells *via* their active and passive targeting effect. The pH-responsive acetalated-CD helped nucleic acid drug anti-miR33 reach the action site effectively, which showed the powerful silencing effect. Overall, AMM NP exhibited considerable anti-atherosclerosis *in vitro* and *in vivo*.

The plaque inflammatory microenvironment is accompanied by overexpressed ROS, which substantially contributes to the advancement of atherosclerosis. ROS scavenging has become a promising anti-atherosclerotic treatment strategy [91, 92]. Wang *et al.* [57] devised a β-CD-based nanoparticle (TPCD NP) for eliminating broad-spectrum ROS. β-CD materials were simultaneously linked with Tempol (Tpl) and phenylboronic acid pinacol ester (PBAP) to form TPCD, which could scavenge a variety of ROS, such as hydrogen peroxide, hydroxyl radical, and superoxide anion. A monolayer of phospholipids, formed by 1, 2-distearoyl-sn-glycero-3-phosphoethanolamine-poly(ethylene glycol) (DSPE-PEG) and lecithin, surrounded the hydrophobic TPCD to form final TPCD NP. The form of core-shell structure was driven by hydrophobic interactions between DSPE-PEG/lecithin and β-CD carrier material. The peripheral PEG chains improved the stability of TPCD NP and facilitated long circulation. TPCD NP, prepared by a facile and well-established nanoprecipitation technique, could target nanotherapy of atherosclerosis and exhibited good anti-oxidation and anti-inflammation effects.

**Figure 4.**
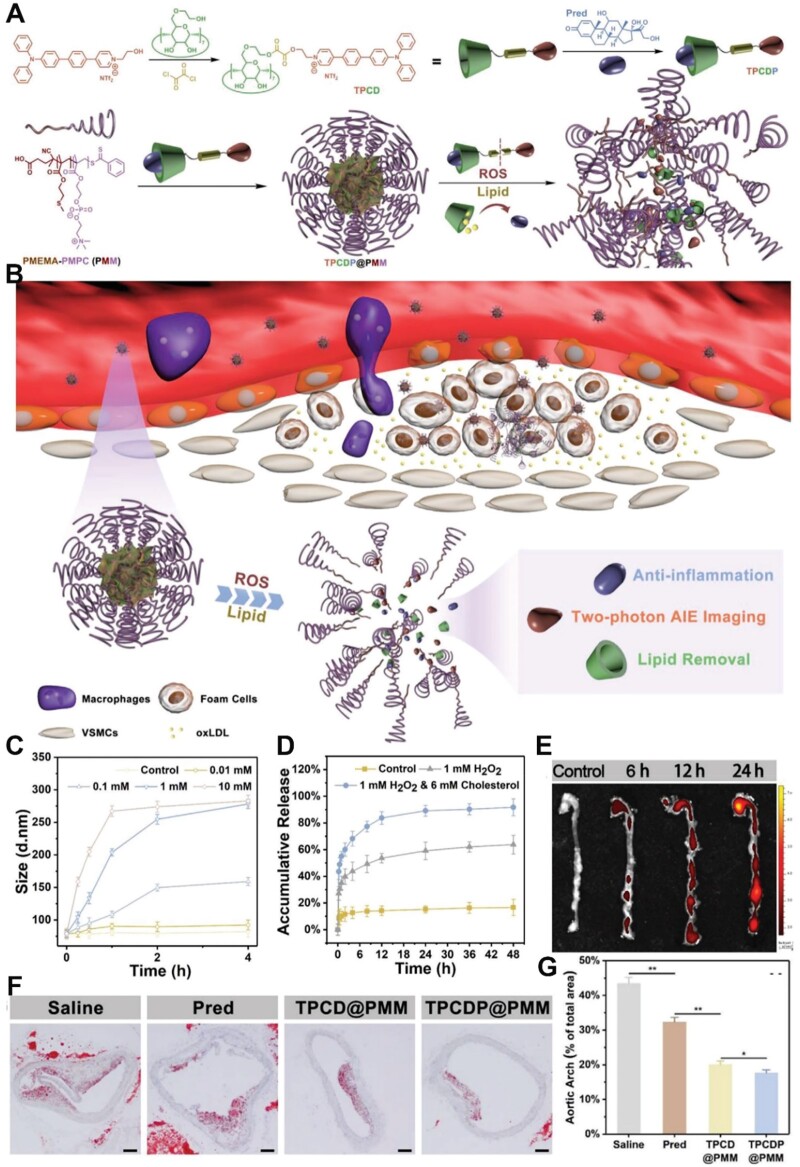
ROS responsive nanoplatform TPCDP@PMM for theranostics of atherosclerosis. (**A**) Illustration of TPCDP@PMM preparation and its reactions to ROS and lipid. (**B**) Illustration of TPCDP@PMM for theranostics. (**C**) Size of TPCDP@PMM under different H_2_O_2_ concentrations. (**D**) Accumulative release of Pred under different treatments. (**E**) Fluorescent images of TPCDP@PMM accumulated in aortas *ex vivo*. (**F**) ORO-stained images and (**G**) quantification of the plaque area treated with different formulations. Adapted with permission from Ref [6].

In addition, over-expressed ROS can trigger the controlled release of drugs [93]. Various ROS-responsive materials have been utilized in the nanotherapy of atherosclerosis [94–96]. Ma *et al.* [6] constructed a ROS-responsive nanoplatform for atherosclerosis theranostics (Figure 4A). The nanoplatform consisted of a core based on two-photon fluorophore-CD/prednisolone complexes (TPCDP) and a polymeric micelle shell based on ROS-responsive amphiphilic poly (2-methylthio ethanol methacrylate)-poly (2-methacryloyloxyethyl phosphorylcholine) (PMEMA-PMPC, PMM) copolymer (Figure 4A). The two-photon fluorophore was connected to CD by a ROS-sensitive bond, and prednisolone (Pred) was carried in the cavities of CD *via* host-guest interaction to form the TPCDP. The final TPCDP@PMM was constructed by wrapping ROS-responsive PMM around the TPCDP. The TPCDP@PMM could pass through the broken vascular endothelium and accumulate in atherosclerosis tissue *via* passive targeting. Subsequently, PMM was disassembled, and TPCDP was released under ROS high level. TPCDP was further broken by the action of the rich ROS and lipid with the release of two-photon fluorophore and Pred (Figure 4B). After H_2_O_2_ treatment, the size of TPCDP@PMM changed obviously (Figure 4C). Moreover, almost 80% Pred was released within 8 h after H_2_O_2_ and lipid treatment, indicating that TPCDP@PMM could achieve drug-controllable release under the specific atherosclerosis microenvironment (Figure 4D). TPCDP@PMM exhibited a strong fluorescent signal in atherosclerosis plaque, indicating the accumulation of nanoparticles and the aggregation-induced emission effect of two-photon fluorophore (Figure 4E). Furthermore, TPCDP@PMM revealed favorable antiatherosclerosis activity with the fewest plaques found in the aortas (Figure 4F and G). Moreover, Ma *et al* [58]. also constructed a β-CD-containing ROS/matrix metalloproteinase (MMP) dual-responsive nanoplatform (PLCDP@PMH) for targeting theranostics of atherosclerosis. Similarly, β-CD, as an important component, undertook a variety of functions, including a lipid solubilizer for treatment, a carrier for bridging liver X receptor ligand T0901317 (LXR-L) and loading Pred, and a core for building nanoplatform. The ROS-responsive polymer PMEMA coating was used in the middle layer of PLCDP@PMH to package the CD complex core and the photoacoustic imaging agent. Finally, the crosslinking products of oxidized hyaluronic acid (oxHA) and an MMP-9 sensitive peptide were coated on the outer layer of the nanoplatform. The nanoplatform demonstrated improved targeting efficacy at atherosclerotic lesions depending on the specific binding of oxHA to the overexpressed CD44 and vascular cell adhesion molecule-1 on the active endothelium. Overexpressed ROS and MMP in atherosclerotic lesions triggered the disintegration of the nanoplatforms and the release of drugs and photoacoustic imaging agents. Pred was replaced by cholesterol from the β-CD cavity, thereby increasing lipid removal and exerting anti-inflammatory effects. Besides, LXR-L upregulated the associated transporters and further enhanced lipid efflux. Furthermore, PLCDP@PMH showed a strong photoacoustic signal in the atherosclerotic area. As a result, the targeted PLCDP@PMH nanoplatform presented encouraging clinical prospects.

**Figure 5.**
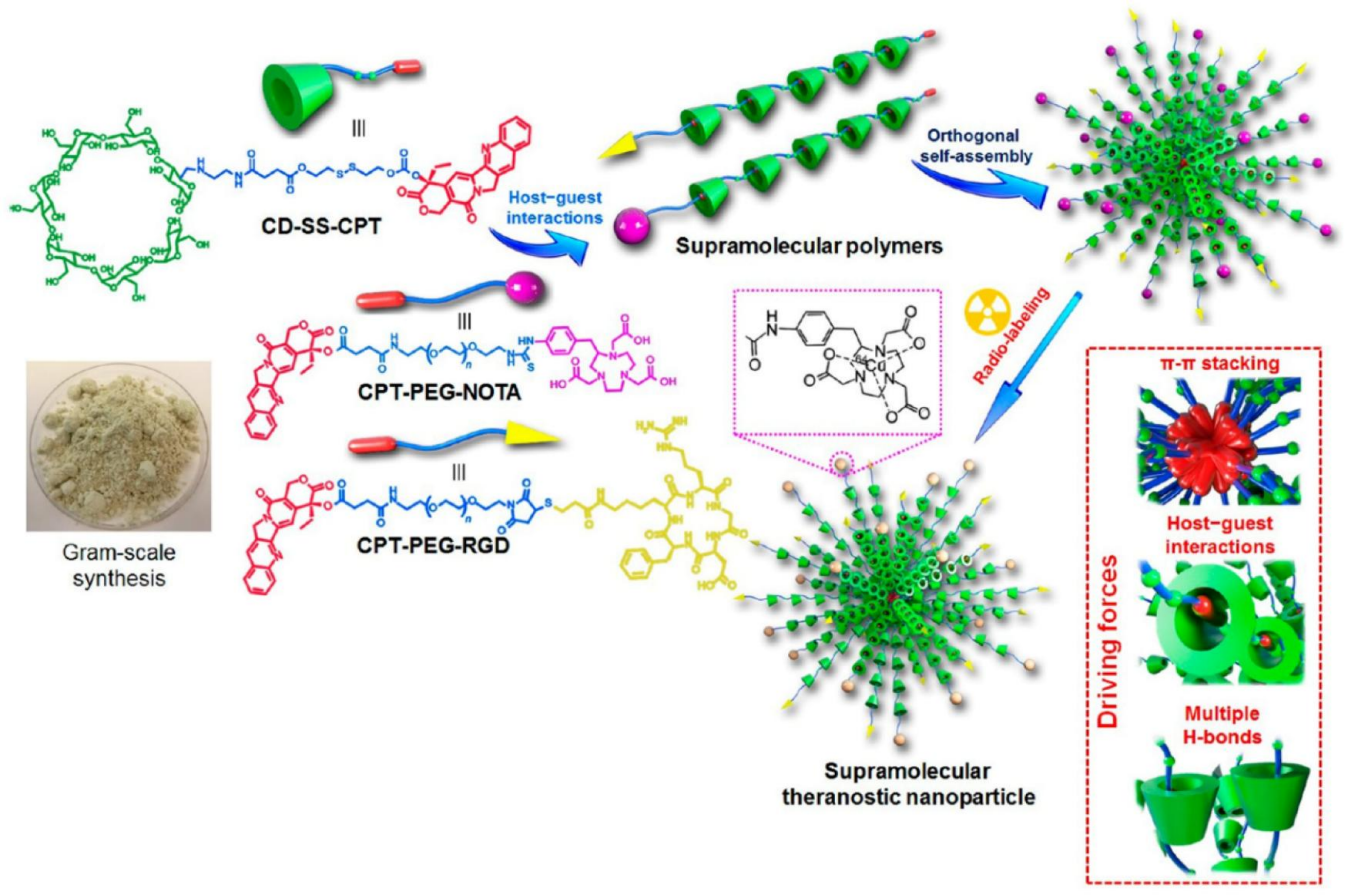
Illustration of supramolecular polymer preparation and supramolecular nanoparticle construction. Adapted with permission from Ref [98].

In particular, supramolecular polymers can be assembled by utilizing the host-guest relationship between CDs and drugs [28, 97–99]. Yu *et al.* [98] engineered a supramolecular polymer, employing β-CD as the host molecule and camptothecin (CPT) as the guest, connected *via* a disulfide bond. The supramolecular nanoparticle was synthesized *via* orthogonal self-assembly mechanisms, facilitated by π−π stacking, host–guest interactions and hydrogen bonds (Figure 5). The supramolecular polymeric nanomedicine exhibited good therapeutic efficacy and provided an ingenious strategy for constructing CD-containing nanoassemblies.

### β-CD/polymer covalent conjugates-based nanoassemblies

There are two basic methods for constructing β-CD/polymer covalent conjugates. One involves coupling the functional group on the modified β-CD with the functional group of the polymer [100–102]. The other method is to use modified β-CD as an initiator to initiate polymerization for forming the CD-polymer conjugates.

**Figure 6.**
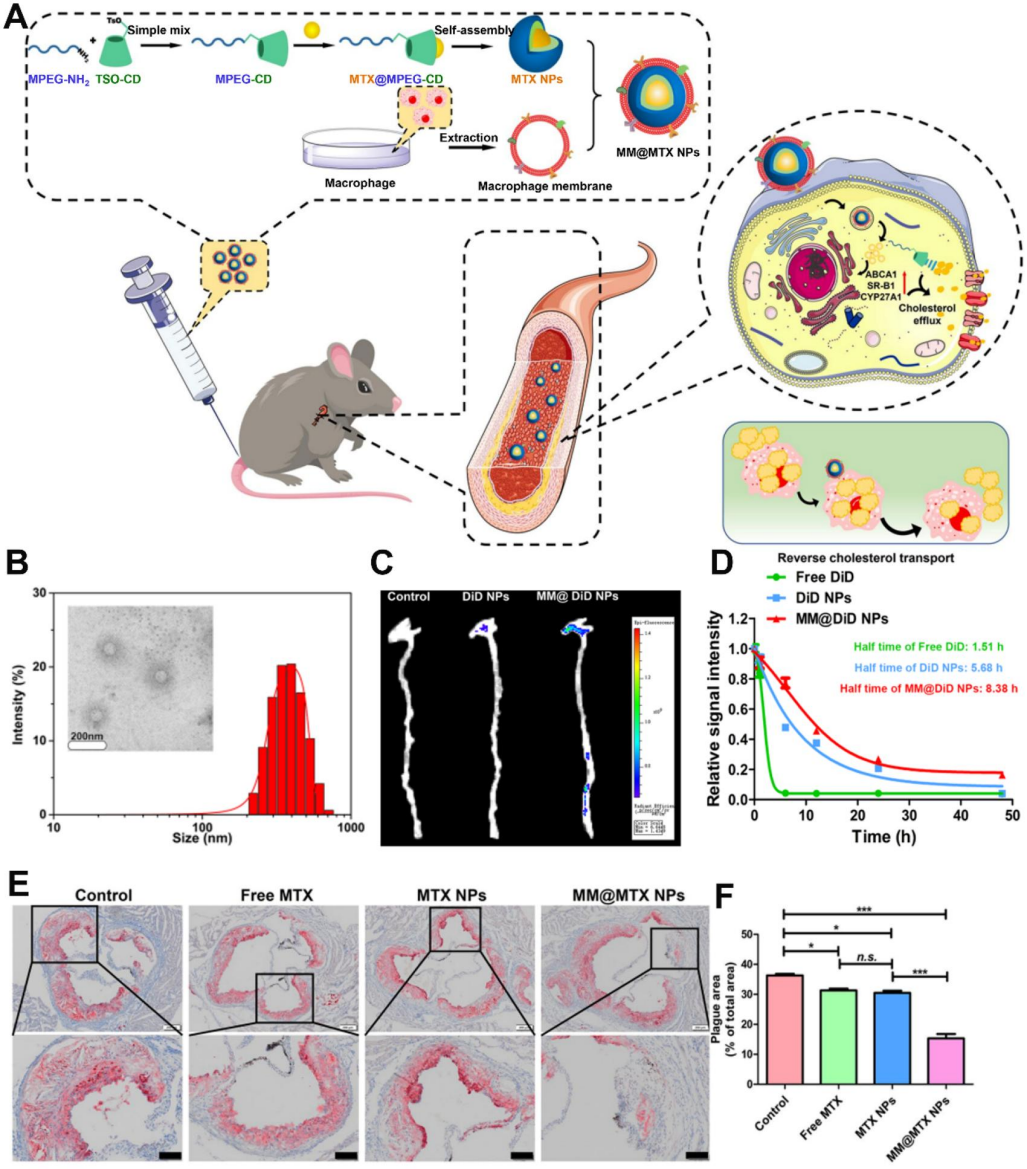
Biomimetic nanoparticles MM@MTX NPs for treatment of atherosclerosis. (**A**) Illustrations of MM@MTX NPs preparation and their treatment for atherosclerosis. (**B**) TEM image and size of MM@MTX NPs. (**C**) Representative fluorescence images displaying the DiD fluorescent signal in the aorta. (**D**) Relative signal intensity of different formulations in blood. (**E**) ORO-stained images and (**F**) quantification of the plaque area treated with different formulations. Adapted with permission from Ref [59].

β-CD has certain hydrophobicity and can be connected with hydrophilic polymer to self-assemble nanoparticles for encapsulating and delivering drugs. Zhu *et al.* [59] synthesized β-CD-p-toluenesulfonyl (CD-OTS) and functionalized CD-OTS with NH_2_-MPEG for MPEG-β-CD. Due to its amphipathic properties, namely the hydrophilicity of PEG and the hydrophobicity of CD, MPEG-β-CD self-assembled to form nanoparticles (MTX NPs) for loading the hydrophobic drug methotrexate (MTX). To target delivery to atherosclerotic plaques, MXP NPs were coated with macrophage membranes (MM) to develop nano-size MM@MTX NPs (Figure 6A and B). The MM coating significantly extended the blood retention time and enhanced drug enrichment in the aorta (Figure 6C and D). MM@MTX NPs with β-CD and MTX could reverse cholesterol transport, suppress the formation of foam cells and significantly reduce the area of plaque (Figure 6E and F). To achieve more efficient and specific coupling, reactivity click chemistry with equimolarity, fast timescale and high yields has also been employed [103–105]. Azido-modified β-CDs, such as mono-(6-azido-6-deoxy)-β-CD, have been coupled with alkyne groups-functionalized polymers [106]. Bohm prepared β-CD-modified hyperbranched PEI *via* a click reaction and self-assembled it into vesicles for drug delivery [107]. Ravishankar *et al.* prepared CD-ferritin nanocage conjugates (AfFtnAA-CD) by copper(I)-catalyzed azide/alkyne cycloaddition reaction. The β-CDs were efficiently chemically conjugated to the external surface of AfFtnAA nanocages. The AfFtnAA-CD could sequester cholesterol from foam cells and exhibit potential use as a therapeutic agent for atherosclerosis [108].

Another approach is to obtain β-CD/polymer covalent conjugates using a range of polymerization techniques such as ring-opening polymerization (ROP) [109, 110], atom transfer radical polymerization (ATRP) [111–114] or reversible addition-fragmentation chain-transfer (RAFT) with β-CD or β-CD derivative as the initiator [21, 115]. Yang *et al.* synthesized thiol-modified β-CD (βCD-SH) as an initiator. They obtained a novel star polymer (βCD-CPD) containing a β-CD core and multiple cell-penetrating poly(disulfide) (CPD) arms through ROP [109]. The nanosystem based on βCD-CPD was capable of simultaneous delivery of the small molecule drug CPT and miRNA *via* the β-CD cavity and charge interaction. Besides, most CD-based ATRP initiators are halogenated β-CDs, such as 2-bromoisobutyryl bromide (BIBB) modified-CD (CD-BIBB) [116–118]. CD-BIBB is usually applied to initiate the ATRP of monomers to obtain polymers with different functionalities [119, 120]. Lin *et al.* [111] prepared a star zwitterionic carboxybetaine polymer *via* ATRP from a CD-BIBB initiator. The CD-carboxybetaine nanoparticles exhibited prolonged circulation time and elicited no immune response, potentially serving as an excellent alternative to PEG for drug delivery systems. Zhu *et al.* [113]s constructed an efficient gene carrier composed of β-CD-BIBB and cationic polymers, including ethanolamine (EA)-functionalized poly(glycidyl methacrylate) (PGEA) and poly((2-dimethyl amino)ethyl methacrylate) (PDMAEMA) using ATRP and click chemistry. Similar to ATRP initiators, CD-coupled RAFT agents have been synthesized. Typically, several RAFT agents with the thiocarbonyl thio group were coupled to β-CD for synthesizing β-CD-RAFT agents. β-CD-RAFT agents initiated polymerization to obtain polymers for constructing multifunctional particles [115]. Nonetheless, the steric hindrance of RAFT agents could limit the synthesis of star polymers. In summary, β-CD can be modified with amino, thio, azido or halogens, which can be further used as attachment points for polymers or as initiators for polymerization.

**Figure 7.**
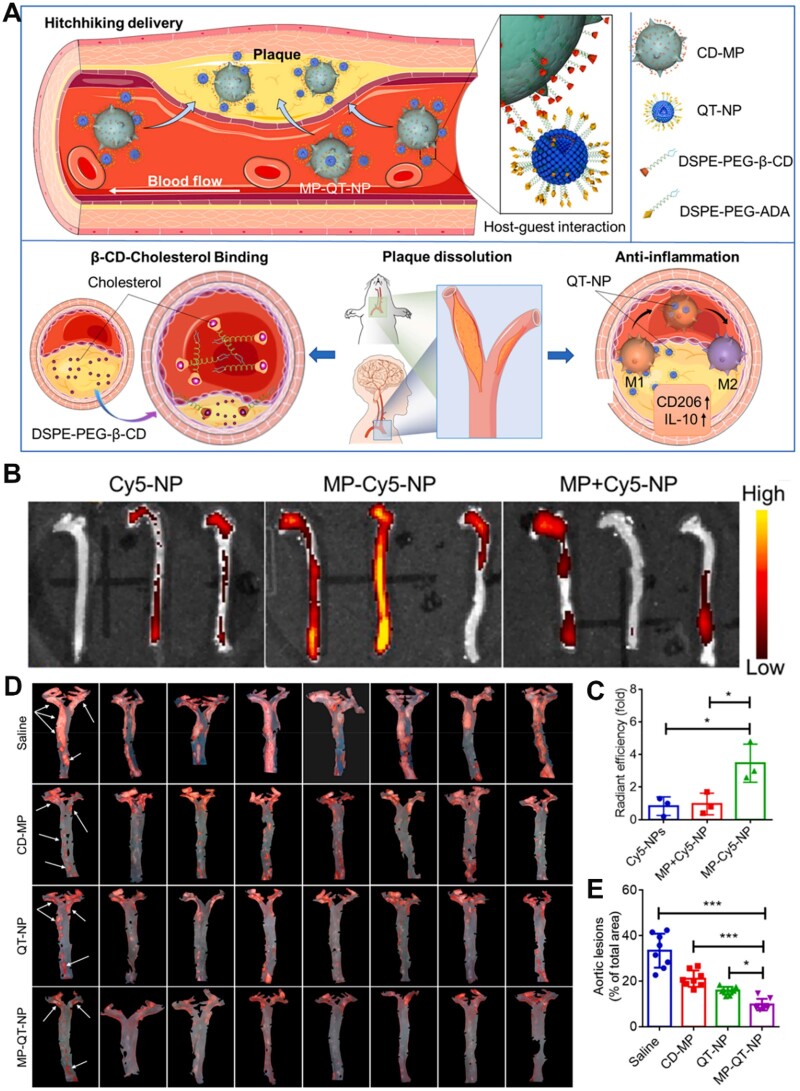
β-CD-mediated macrophage-liposome conjugate for targeted anti-atherosclerosis delivery and therapy. (**A**) Schematic illustration of MP-QT-NP-mediated hitchhiking delivery and treatment for atherosclerosis. (**B**) Fluorescence images and (**C**) quantitative analysis of different formulations accumulation in aorta. (**D**) Microscope images of aortic lesions and (**E**) quantitative analysis of aortic lesions. Adapted with permission from Ref [123].

The interactions between β-CD and polymer often include both covalent and non-covalent bonds. In particular, the special cavity of β-CD can provide binding sites for polymers with a suitable structure [121]. Zhang *et al.* constructed nanoassemblies through supramolecular assembly with β-CD and polymers *via* covalent bonding and host-guest interaction. CDI-modified β-CD was firstly prepared for coupling poly-[(*N*-2-hydroxyethyl)-aspartamide]-Pt(IV) (PHEA-Pt(IV)), thereby synthesizing the assembly module (PHEA-Pt(IV)/β-CD, PPCD). Subsequently, the nanoassemblies were constructed by one-pot supramolecular assembly *via* host-guest interactions between β-CD in PPCD and adamantane (ADA) in other assembly polymer modules [122]. Taking into account the pathological features of macrophage recruitment in plaque formation and progression, targeted therapy for atherosclerosis was achieved through the construction of macrophage hitchhiking delivery systems utilizing host-guest interactions [45]. Gao *et al.* [123] constructed a CD-mediated macrophage-liposome conjugate for targeted anti-atherosclerosis therapy. In detail, β-CD-NH_2_ was coupled to DSPE-PEG-NHS *via* an amidation reaction for preparing DSPE-PEG-β-CD. Then, DSPE-PEG-β-CD was inserted into the macrophage to obtain the β-CD-modified macrophage (CD-MP) (Figure 7A). In addition, liposome loaded with quercetin (QT-NP) was formulated, and the surface of QT-NP was modified with ADA. The CD-MP and QT-NP were mixed to prepare the macrophage hitchhiking delivery system (MP-QT-NP) *via* host-guest interactions (Figure 7A). Compared to the Cy5-NP and MP+QT-NP groups, ApoE^−^/^−^ mice treated with MP-QT-NP exhibited the strongest signals in the aorta, approximately four times that of the other groups (Figure 7B and C). The findings suggested that MP-QT-NP conjugate enhanced targeted delivery to plaque *via* host–guest interactions between β-CD and ADA. As shown in Figure 7D and E, the aortic lesion area decreased to 18.3% in the QT-NP group, whereas mice in MP-QT-NP group observed the lowest aortic lesion area at 8.6%. This reduction was attributed to the targeted delivery facilitated by macrophage-hitchhiking to atherosclerotic plaques, mediated by host–guest interactions between β-CD and ADA. Additionally, the binding of β-CD-cholesterol and the anti-inflammatory effects of QT-NP within the plaques further contributed to this observed improvement.

In the process of constructing nanoparticles, β-CD and polymers engage in various interactions, including covalent bonding, host–guest interactions, hydrogen bonding, van der Waals forces and other non-covalent interactions. The specific forces involved can vary depending on the types of β-CD, guest molecules and polymers used in the formulation. The combination of these interactions between β-CD and polymers leads to the formation of stable nanoassemblies, which can have various applications in targeted drug delivery, controlled release systems, integration of diagnosis and treatment and other nanotechnology-related fields.

### Poly(β-CD)-based nanoassemblies (1000)

#### The pharmacokinetics of poly(β-CD)-based nanoassemblies

**Figure 8.**
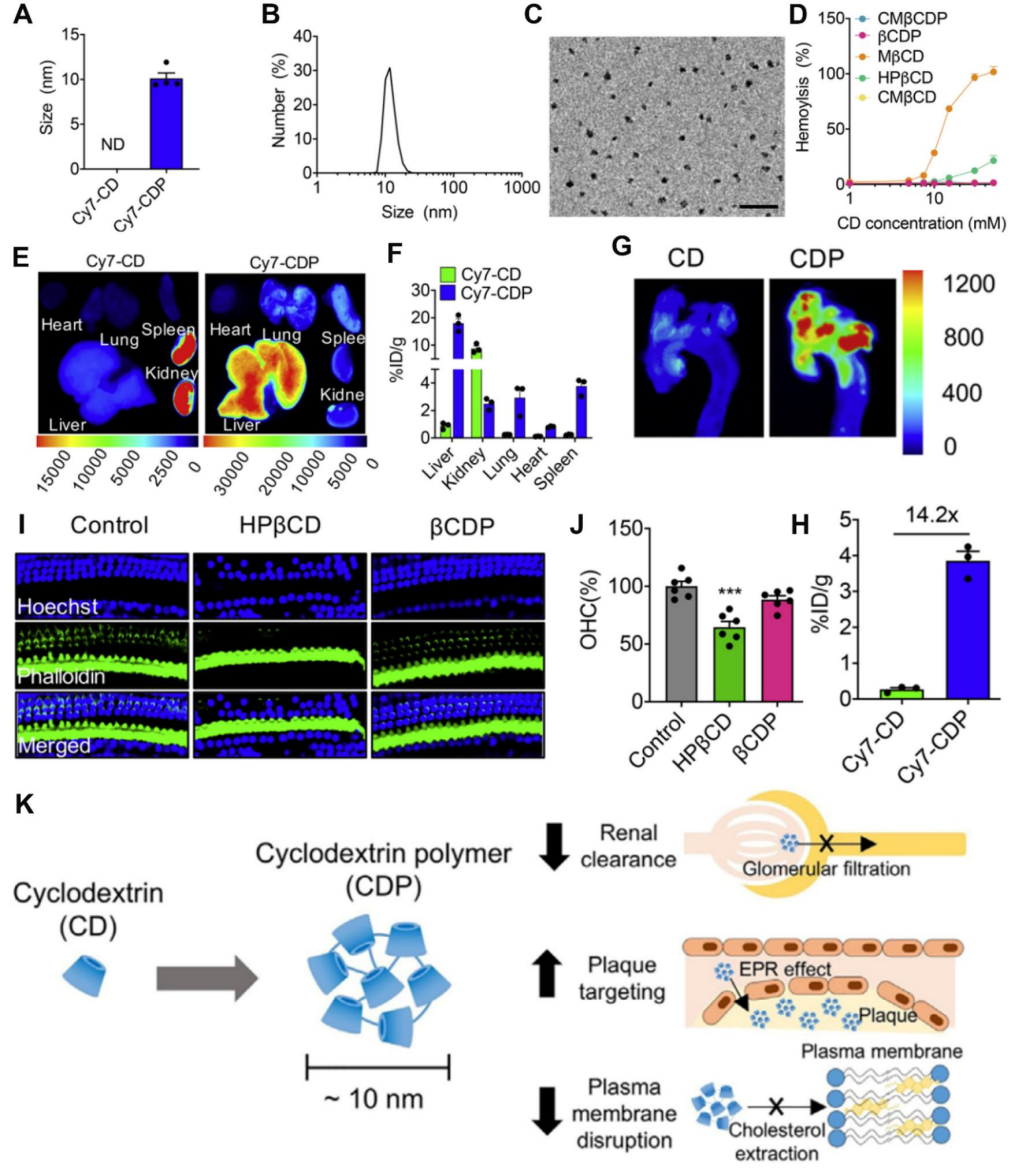
CD polymers CDP for treatment of atherosclerosis. (**A**) Hydrodynamic sizes, (**B**) distribution and (**C)** TEM image of Cy7-CDP. (**D**) Hemolysis of various formulations with different CD dose. (**E**) Representative fluorescence images and (**F**) fluorescence quantification of the dissected organs *ex vivo*. (**G**) Representative fluorescence images and (**H**) quantification of the dissected aorta *ex vivo*. (**I**) Images and (**J**) quantification of Cochlea’s outer hair cells. (**K**) CDP for safe and efficient atherosclerosis treatment. Adapted with permission from Ref [60].

Increasing evidence has indicated that β-CD, as a type of bioactive nanomaterial with anti-inflammatory and cholesterol-lowering effects, can be used as a potential anti-pharmaceutical agent against atherosclerosis [124–126]. While β-CD and its derivatives exhibit promising therapeutics for atherosclerosis, their individual use is limited due to poor pharmacokinetics and ototoxicity of CD molecules [127]. A study demonstrated that β-CD polymer (poly(β-CD)) possessed a stronger cholesterol-dissolving ability and drug-loading ability compared with that of β-CD [61]. Moreover, poly(β-CD) can improve pharmacokinetics and reduce ototoxicity compared to CD [128]. Kim *et al.* [60] synthesized Cy7 fluorescent dye-conjugated CD polymers (Cy7-CDP) or CD (Cy7-CD) in order to explore whether CDP displayed enhanced pharmacokinetics compared to CD monomer. The Cy7-CDP showed a hydrodynamic size of 10.1 ± 0.6 nm, while Cy7-CD showed no nano-size (Figure 8A–C). Notably, all types of CD monomers exhibited hemolytic activities, while β-CD polymer showed no hemolysis (Figure 8D). It may be that the arrangement and dimension of the poly(β-CD) restricted the availability of CD to cholesterol located in the cell membrane. After intravenous injection, Cy7-CDP exhibited a half-life of 26.8 h, about 58 times that of the CD. According to the Figure 8E and F, Cy7-CD was primarily distributed in the kidney, while Cy7-CDP was primarily removed in the liver. These findings demonstrated that the pharmacokinetics of the CDP were significantly improved, and the biodistribution was notably altered, which was due to the fact that particles larger than 8 nm could greatly avoid being filtered by the kidney [129]. Figure 8G and H showed that the dissected aorta in Cy7-CDP had a fluorescence intensity that was 14.2 times stronger than that of Cy7-CD, suggesting that the nano-sized CDP could improve plaque accumulation by enhanced permeability and retention effect. The CDP significantly inhibited plaque growth and exhibited good therapeutic effects for treatment of atherosclerosis. Ototoxicity caused by CD damage to the plasma membrane by CD is an important reason limiting its application. As shown in Figure 8I and J, the mice treated with poly(β-CD) βCDP showed no ototoxicity. In summary, CDP improved plaque targeting reduced nephrotoxicity and ototoxicity compared to CD monomer (Figure 8K), enabling poly(β-CD)-based nanotherapy to be a safe and effective option for atherosclerosis.

#### The reduction of CCs by poly(β-CD)-based nanoassemblies

**Figure 9.**
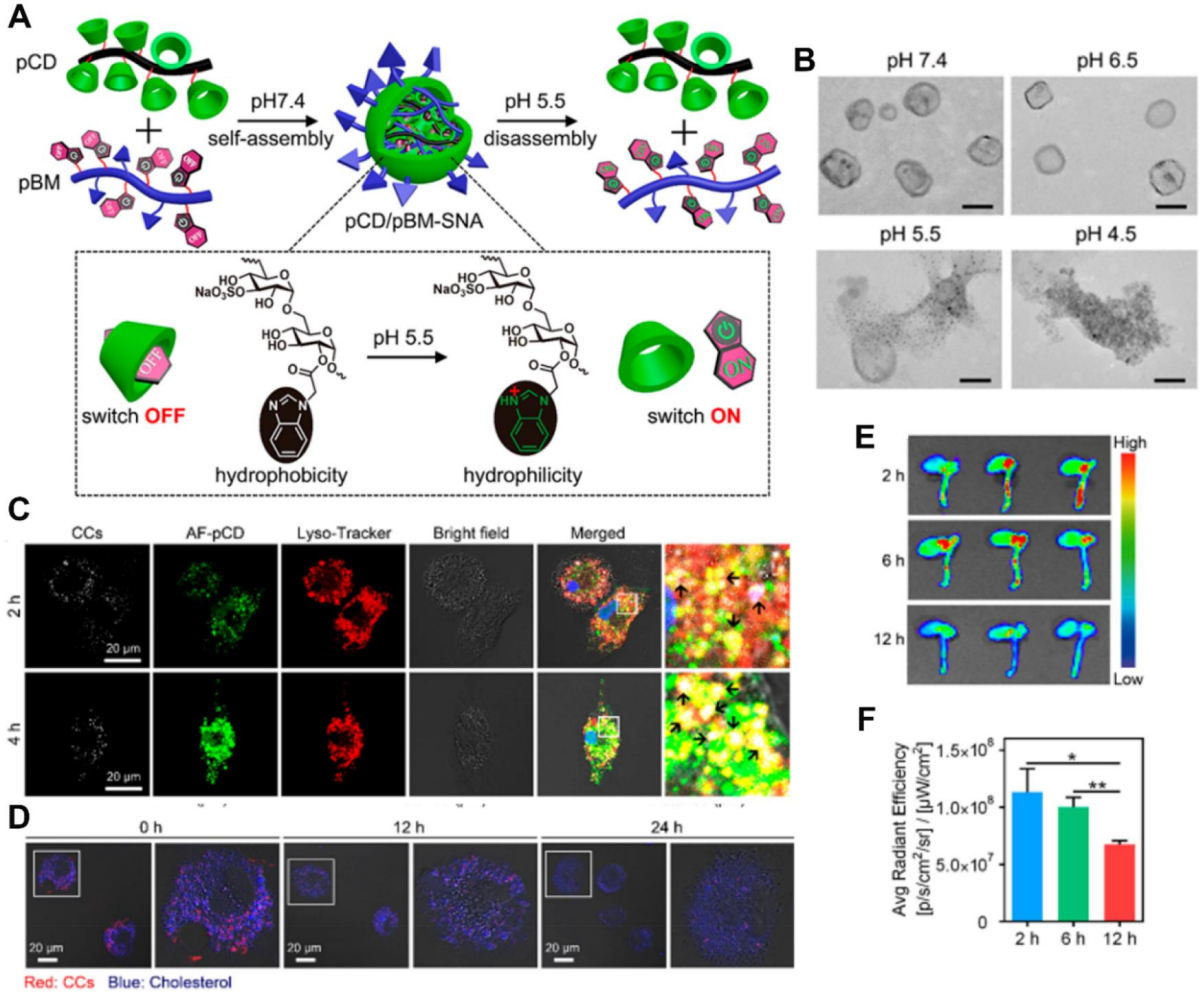
pCD supramolecular nanoassembly pCD/pBM-SNA for anti-atherosclerosis. (**A**) Schematic diagram showing how pCD/pBM-SNA self-assemble and disassemble at different pH levels. (**B**) TEM images of nanoassembly at different pH values. (**C**) Colocalization images of pCD/pBM-SNA and lysosomes. (**D**) Removal of the cholesterol and lysosomal CCs. (**E**) The image and (**F**) fluorescence quantification of accumulation of nanoassembly in the heart and aorta *ex vivo*. Adapted with permission from Ref [61].

It is well recognized that CCs are essential to the development of atherosclerosis. Research has demonstrated that CCs initially form and gather in the lysosomes of cells high in cholesterol [61, 130]. However, the massive CCs in the lysosomes can promote the formation and apoptosis of foam cells, further leading to inflammation and plaque formation and development [130–132]. Therefore, it is crucial to clear and remove lysosomal CCs from macrophages or foam cells in order to treat atherosclerosis. It is worth noting that the lysosomal microenvironment is acidic with a pH of 4–6, so pH-sensitive materials have been widely used for drug delivery and therapy related to lysosomal-related diseases [96, 133–136]. Zhang and colleagues [61] engineered a supramolecular nanoassembly (pCD/pBM-SNA) by using multivalent inclusion interactions between poly β-cyclodextrin (pCD) and poly-benzimidazole (pBM) to self-assemble (Figure 9A). Under the physiological condition (pH 7.4), the association constant between hydrophobic BM and the CD cavity was strong. Once in the acidic microenvironment (pH 4–6), BM would be protonated, and its hydrophobicity would become hydrophilic [137, 138], thus separating pBM from pCD, disintegrating the nanoassembly and exposing the CD cavity to remove lysosomal CCs. Figure 9B displayed that the morphological changes of the nanoassembly pCD/pBM-SNA at different pH values. Under the physiological condition (pH 7.4), the nanoassembly showed nanoparticle morphology with a size of 60 nm. As the pH decreased to 5.5, the supramolecular nanoassembly began to disassemble, and disintegrated completely at pH 4.5. Those revealed that the nanoassembly was pH-sensitive. Images in Figure 9C showed the intracellular fate of pCD/pBM-SNA. After co-culture for 4 h, the nanoassembly (green) and lysosomes (red) showed obvious co-localization, indicating the possibility of pCD/pBM-SNA internalization *via* the endocytosis-endosome/lysosome pathway. The considerable separation of pCD and pBM after a further 6 h suggested that pCD/RhB-pBM-SNA could dissociate in the endosome/lysosome as expected. With the extension of culture time, the nanoassembly greatly reduced cholesterol and lysosomal CCs (Figure 9D). Furthermore, pCD/pBM-SNA exhibited an excellent plaque-targeting effect, which was attributed to the fact that dextran sulfate could actively target atherosclerotic plaque (Figure 9E and F). The pH-sensitive nanoassembly showed good biocompatibility and therapeutic effect.

β-CD can form complexes with many hydrophobic drugs through host–guest interactions. Similarly, multivalent polymer-polymer complexes have been formed between poly(β-CD) and polyprodrugs with suitable structures [139–142]. These complexes tend to yield strong nano-assemblies with high stability, which can significantly improve the solubility of hydrophobic drugs in the aqueous solutions and achieve effective drug delivery [139]. Namgung *et al.* constructed a nanoassembly based on host-guest interactions between polymer CD and polymer paclitaxel. The nanoassembly efficiently delivered drug into the target cells and exhibited significant therapeutic effects [140].

The β-CD derivatives-based, β-CD/polymer-based and poly(β-CD)-based drug delivery systems differ mainly in their composition and structure, resulting in differences in their properties and uses in drug delivery. β-CD derivatives are recognized for their capacity to selectively solubilize hydrophobic drugs by forming inclusion complexes. This selectivity is dependent on factors such as the dimensions, configuration and polarity of the drug molecule, in addition to the substituents present on the β-CD derivatives [26, 124]. Drug selectivity in β-CD/polymer-based systems may be impacted by the polymer selection. Drug molecules may interact differently with polymers that have particular functional groups or topologies, thereby influencing their encapsulation efficiency and release kinetics. Furthermore, the β-CD/polymer-based systems with microenvironment-sensitive groups often offer controlled release of drugs in specific environments, and some β-CD/polymer-based systems with targeting moieties may integrate targeting moieties to improve selectivity towards specific cells or tissues [58, 99]. Poly(β-CD) structures, with multiple β-CD units, may exhibit enhanced drug-binding capacity in contrast to monomeric β-CD derivatives. This multivalency effect can improve selectivity by accommodating multiple drug molecules simultaneously [24, 97]. In summary, the specific design and composition of each system can significantly influence its selectivity profile.

## Conclusions and perspectives

This review has summarized and discussed β-CD-based nanotherapies for anti-atherosclerosis. Notably, the unique structure and desirable properties of β-CD could facilitate dual functions of treatment and drug delivery in the field of atherosclerosis. On the one hand, the antioxidative and anti-inflammatory properties of β-CD or its derivatives have been used in the treatment of inflammatory diseases, such as cardiovascular and CVDs, cancers, peritonitis and acute lung injury [26, 124, 143, 144]. On the other hand, the special structure of β-CD with a cavity can bind cholesterol and CCs, hydrophobic drug molecules and many functional modules. Studies indicated that β-CD or β-CD derivatives could bind with hydrophobic drugs or polymers with hydrophobic structures to form stable and strong nanoassemblies. These nanoassemblies could improve drug solubility in aqueous solution, achieve drug-effective delivery, enable drug cholesterol-sensitive release and enhance atherosclerotic drug therapy [17, 51]. Moreover, decoration with specific targeting units or coating with leukocytes and macrophage membranes could notably improve plaque-targeted delivery of β-CD-based drug delivery systems [123, 145–147].

With the development of CDs and nanotechnology, nanoassemblies based on β-CD and its derivatives have been used for drug delivery [54, 66, 82]. In this review, β-CD derivatives-based, β-CD/polymer-based and poly(β-CD)-based drug delivery systems were summarized. Although β-CD and its small molecular derivatives, such as SBE-β-CD, M-β-CD and HP-β-CD, have been successfully used as pharmaceutical excipients, there are still many problems to be overcome in the CD-based nanotherapy for clinical studies [18]. Firstly, although partial β-CD derivatives have exhibited good biocompatibility, the biosafety of additional functional components used to prepare nanoassemblies remains to be investigated. CDs that introduced other functional groups in the references have been validated at the laboratory level or in mouse studies, further clinical safety assessments are necessary to ensure their safety for human use [6, 54, 59, 94]. Secondly, generally speaking, the more complex the modification, the higher the cost. Therefore, simple and effective synthetic approaches and production processes are necessary to improve production efficiency, reduce production cost and improve the quality and stability of β-CD-based nanoassemblies. Thirdly, studies on the fate *in vivo* of CD-based nanoassemblies are still insufficient. Clinical research requires the transition of nanoassemblies from the laboratory to the practical application, and studying their fate *in vivo* is a key link in this process. Further research is also needed on the long-term safety and efficacy of β-CD-based nanoassemblies *in vivo*. Despite the challenges in clinical applications mentioned above, β-CD-based nanoassemblies will evolve with the development of chemistry, nanotechnology, nanomedicine, pharmaceutics and nanotoxicology, having a significant impact on the management of atherosclerosis.
